# The Role of Molecular Imaging as a Marker of Remyelination and Repair in Multiple Sclerosis

**DOI:** 10.3390/ijms23010474

**Published:** 2021-12-31

**Authors:** Ido Har-Shalom, Arnon Karni, Hadar Kolb

**Affiliations:** 1 Department of Neurology, Tel Aviv Sourasky Medical Center, Tel Aviv-Yafo 6423906, Israel; ido.ben-shalom@weizmann.ac.il (I.H.-S.); arnonk@tlvmc.gov.il (A.K.); 2 Department of Neurobiology, Weizmann Institute of Science, Rehovot 76100, Israel; 3 Sackler Faculty of Medicine, Tel Aviv University, Tel Aviv-Yafo 6423906, Israel; 4 Sagol School of Neuroscience, Tel Aviv University, Tel Aviv-Yafo 6423906, Israel

**Keywords:** multiple sclerosis, neurodegeneration, neuroprotection, remyelination, disease progression, molecular biomarkers, positron emission tomography (PET), magnetic resonance spectroscopy (MRS), sodium imaging

## Abstract

The appearance of new disease-modifying therapies in multiple sclerosis (MS) has revolutionized our ability to fight inflammatory relapses and has immensely improved patients’ quality of life. Although remarkable, this achievement has not carried over into reducing long-term disability. In MS, clinical disability progression can continue relentlessly irrespective of acute inflammation. This “silent” disease progression is the main contributor to long-term clinical disability in MS and results from chronic inflammation, neurodegeneration, and repair failure. Investigating silent disease progression and its underlying mechanisms is a challenge. Standard MRI excels in depicting acute inflammation but lacks the pathophysiological lens required for a more targeted exploration of molecular-based processes. Novel modalities that utilize nuclear magnetic resonance’s ability to display in vivo information on imaging look to bridge this gap. Displaying the CNS through a molecular prism is becoming an undeniable reality. This review will focus on “molecular imaging biomarkers” of disease progression, modalities that can harmoniously depict anatomy and pathophysiology, making them attractive candidates to become the first valid biomarkers of neuroprotection and remyelination.

## 1. Introduction

Multiple sclerosis (MS) is an inflammatory autoimmune demyelinating disease of the central nervous system (CNS). Pathognomonic to MS are focal lesions in the white matter, which are referred to as plaques. These are demyelinated foci with variable degrees of inflammation, gliosis, and neurodegeneration. Lesions can be found throughout the CNS, with known predilections to the optic nerve, cortical/juxta cortical, paraventricular white matter, infratentorial, and spinal cord region [1]. On MRI, lesions are formally defined as a hyperintense focus on T2-weighted (T2w) sequences. Typical MS lesions are demarcated round or ovoid foci, at least 3 mm on their longest axis [2,3].

The disease has a complex pathophysiology that comprises inflammation, demyelination, neuroaxonal degeneration, repair, and remyelination at various degrees [4]. BBB (blood–brain barrier) disruption is an early event in the inflammatory response seen in MS, mainly involving small-to-medium-sized veins. Followed by this is an influx of blood-derived inflammatory cells into the tissue, activation of macrophages/microglia, oligodendrocyte loss, and striping of axons from myelin (i.e., demyelination) throughout the lesion [5]. For most MS lesions, inflammation clears out with time, and lesions can either undergo spontaneous remyelination (i.e., repair of the damaged myelin sheath) or remain fully demyelinated [6]. In a subset of lesions, inflammation persists in the chronic stage, primarily composed of activated microglia/macrophage concentrations at the lesion’s edge [7]. Unique to MS is the relative preservation of axons and neurons in newly formed lesions. However, neuronal axon loss (i.e., neurodegeneration) is present in all chronic lesions, although considerable variability exists [8]. The relative prominence of each underlying mechanism varies between patients and within the same patient at different times, contributing to the biological heterogeneity of the disease.

Corresponding to the diverse pathophysiology, clinical presentation and progression in MS vary [9]. For most MS patients, the disease starts with waves of inflammatory demyelination termed relapses followed by spontaneous recovery. In many cases, some degree of a slow progressive accumulation of disability exists as well. This insidious progression that is predominantly independent of inflammatory relapses is known as “silent MS” and it is this silent progression that dictates long-term outcomes of patients [10].

Acute inflammatory episodes associated with edema and demyelination are characteristic for the early stages of MS but only weakly correlate with long-term progression [11]. In contrast, chronic inflammation is mainly compartmentalized in the CNS and is believed to play a critical role in initiating and propagating neurodegeneration and remyelination failure [12,13]. Unfortunately, current-day therapeutics focus mainly on preventing acute inflammation, lacking the ability to slow down the main drivers of MS progression. The capacity to monitor remyelination and assess neuroprotection in a clinical setting is an essential first step in gauging prognosis and supporting the search for new therapeutic targets for repair.

Biomarkers serve as a tool for disease diagnosis and tracking, helping clinicians to better understand patients’ status, estimate progression, and guide treatment. In MS, biomarkers play an important role in disease diagnosis and in monitoring inflammatory activity. Magnetic resonance imaging (MRI) is the most established biomarker in MS and one of the mainstays of disease diagnosis. This is portrayed powerfully by its prominent part in the McDonald diagnostic criteria [14]. These criteria require evidence of typical damage (i.e., MS lesions) that has occurred in at least two instances and in two or more areas of the CNS, also referred to as dissemination in time (DIT) and space (DIS). Historically, DIT and DIS criteria were met solely on clinical grounds. However, with the introduction of MRI, radiological evidence has been gradually accepted as a substitute for clinical presentation, enabling MS diagnosis after a single clinical event suggestive of MS.

Together with MRI, the latest revisions to MS diagnostic criteria positioned oligoclonal bands (OCB), a biological biomarker, as the second validated diagnostic marker after MRI, proclaiming that their presence in the CSF is enough for establishing DIT for the diagnosis of MS [14]. Trading a molecular biomarker for one of the two pillars of MS diagnosis indicates the significant role OCBs hold today. 

MRI is a biomarker prototype with a good sensitivity and specificity for MS diagnosis and monitoring inflammatory activity, and OCBs are a proven tool for disease diagnosis. However, their capacity to evaluate silent disease progression is limited. Standard MRI generally provides global markers, such as lesion volume and brain atrophy, lacking the ability to present data on the molecular level. Therefore, MRI shows a low specificity for pathophysiologic phenomena, such as remyelination or neurodegeneration. For these mechanisms of disease progression, a molecular biomarker may be better suited. 

Advanced MR modalities utilize various imaging attributes to indirectly assess different pathophysiological processes. Diffusion tensor imaging (DTI), an MRI sequence alternate to diffusion-weighted imaging (DWI), relies on tissue water diffusion to produce images. DTI provides information on the tissue type, components, structure, and integrity based on three-dimensional molecular diffusion parameters in the tissue. Damage to myelin correlates to a change in diffusivity, which relates to disease progression, clinical status, and, in several cases, to pathology. Diffusion-based modalities such as DTI have become widespread, and are arguably the most commonly used proxy for myelin content today in clinical trial settings [15]. However, diffusion parameters are not specific to myelin or axonal pathology and are highly affected by inflammation and edema. A comprehensive meta-analysis reviewing quantitative MRI measures found that diffusion-based measures have the lowest histology correlation compared to all other modalities in this review. Indeed, DTI’s low specificity to molecular changes in the tissue still holds it back from becoming a robust tool for assessing tissue integrity and repair [16].

Promising markers from recent in vivo trials show encouraging results to fill this clinical void. Plasma levels of neurofilament light chains (NfL) hold a high correlation with MRI activity, risk of future lesions, relapses, and an accelerated brain volume loss [17]. Likewise, in vivo molecular imaging approaches with a higher specificity to important pathological substrates see constant development. This includes several promising positron emission tomography (PET) tracers for myelin, inflammation, and neurodegeneration, MR spectroscopy (MRS), and advances in sodium channel MRI (NaMRI). These “molecular imaging” modalities can harmoniously depict anatomy and pathophysiology, making for an appealing myelin and neuroaxonal integrity biomarker candidate. This review will focus on leading molecular imaging biomarkers of neuroprotection and remyelination. Our discussion will focus on these markers that, in our view, have the most vital foundation to connect promising research with a novel clinical toolbox.

## 2. PET

The need for a specific in vivo assessment of the underlying processes characterizing disease progression in MS is continuously eclipsed by the effort to develop novel therapeutics that slow down the clinical decline. MRI is an excellent tool for capturing structural changes, but we can only infer the underlying pathological processes based on them. Positron emission tomography (PET) is a non-invasive, quantitative technique that uses radiolabeled compounds (PET tracers) to characterize various pathological processes. By binding to specific targets, PET imaging can depict metabolic, biochemical, or cellular changes depending on the tracer in use. It mainly pertains to assessing remyelination and microstructural damage, two processes that are hard to discern using conventional structural imaging. Using radiotracers that target these processes to differentiate between potentially reversible demyelination and mostly irreversible neuronal–axonal loss is an essential first step in estimating repair potential in MS.

### 2.1. Demyelination

Pathognomonic for MS is the presence of focal demyelinated lesions, accompanied by a varying degree of neuroinflammation, degeneration, and gliosis. Demyelination and axonal loss are closely related but not interchangeable, the latter being one of the factors affecting remyelinating potential. Growing evidence suggests that long-term clinical disability correlates with higher levels of demyelination and that successful repair may contribute to neuroprotection and improved long-term clinical outcomes.

#### 2.1.1. Specific Quantification of Myelin Is Feasible with Various PET Tracers

The inherent specificity of PET tracers to their target is particularly promising for the direct assessment and quantification of myelin compared to other imaging methods. Since its first introduction in 2006, several stilbene and benzothiazole PET tracers have been shown to selectively bind to myelin, although their specific molecular targets are not well characterized [18,19]. The most studied tracer in this realm has been Pittsburgh Compound B (PiB), initially developed to image amyloid deposition in neurodegenerative disorders and dementia [20]. In recent years PiB’s usage has expanded to in vivo myelin imaging in humans. Similar to other amyloid tracers, PiB binds proteins with aggregated beta-sheet structures. These are present in β-amyloid and myelin basic protein (MBP), explaining the tracers’ high sensitivity as white matter (WM) integrity biomarkers. On MRI, MS lesions that appear as WM hyperintensities (WMH) include fragmented myelin, where the beta-sheet structure of the MBP is lost, and so binding to the tracers diminishes.

#### 2.1.2. Sensitive Characterization of Myelin Kinetic with PET Tracers Helps to Assess Remyelination

Stankoff et al. were the first to show that PiB selectively binds to myelin in both in vitro and in vivo models, including postmortem brain models [21]. PiB was able to separate myelinated tissue from demyelinated tissue. MS lesions showed minimal PiB uptake compared with non-demyelinated WMHs, a potential tool for differentiating MS from non-MS brain lesions. The PET-tracers uptake was also reduced in MS lesions and normal-appearing white matter of MS patients compared to the uptake in the healthy control, reflecting various degrees of demyelination [22,23,24,25]. 

The relative decrease in PiB uptake was lower in contrast-enhancing lesions than in nonactive lesions in a small lesion sample, a proof of concept that quantification and myelin change can be captured with PET imaging [21]. The dynamics of PiB also reflected the myelination status—appearing lower in demyelinated lesions and then correcting to the control-level upon remyelination [26,27]. Overall, PiB’s distinctive uptake dynamics make it specific for MS patients compared to several other biomarkers [28,29], supporting its utility as a biomarker of remyelination in MS.

#### 2.1.3. Imaging Remyelination Outside of MS Lesions

Comparing PET-MRI to other modalities using PiB shows that PET-MRI might be more sensitive than DTI and other conventional MRI sequences in detecting early demyelination [30]. Apart from its role as a direct quantifier of the myelin status, PiB can also depict a more global CNS turnover by quantifying its clearance rate from the CSF. PiB exits the CSF of MS patients more gradually when compared to healthy controls, and this remains true when looking at other CNS diseases or the elderly in general [31].

#### 2.1.4. Novel Myelin-PET Tracers

Apart from the carbon-based benzothiazole derivative PiB, other PET tracers have been explored as myelin markers. A comparison between [N-methyl-11C]-4,4′-diaminostilbene (MeDAS), case imaging compound (CIC), and PiB showed that they behave similarly as myelin tracers. However, MeDAS might have an advantage as a tracer for myelin because it shows a higher brain uptake when compared to PiB [26].

Novel fluorinated PET tracers are characterized by better kinetics, with a longer half-life and higher uptake into CNS tissue, eliminating the need for a cyclotron. This has marked them as strong candidates with the potential to serve as real-world clinical surrogates for myelin assessment [32]. Several studies have demonstrated F-florbetapir’s ability to assess demyelination in MS patients [24,32,33,34]. Recently, Zhang et al. carried out the first longitudinal study examining F-florbetapir’s ability to monitor myelination status in MS, and showed promising results. F-florbetapir is less affected by lesion size and successfully differentiates between demyelination and inflammatory edema, usually indistinguishable on MRI. Moreover, the Expanded Disability Status Scale (EDSS) measuring MS-related disability and myelin changes was correlated with F-florbetapir but not with PiB [33]. This marks F-florbetapir as a promising tool for the future monitoring of the myelin status in MS patients [35].

#### 2.1.5. Tightening the Clinical–Pathological Correlation between Remyelination and Clinical Outcomes

The robustness of myelin-PET tracers as a marker of remyelination is useful for investigating the correlation between remyelination and various clinical outcomes in MS patients. A low PiB uptake in MS lesions and normal-appearing white matter (NAWM) was correlated with a lower performance in visuospatial, memory, language, and other cognitive functions [23,30,36]. Using a long-term follow-up of PiB uptake, Bodini et al. generated a “remyelination index” and successfully correlated it to several clinical scores, showing a direct relationship between PiB, remyelination, and the clinical performance. Accordingly, the lowest scores were calculated for lesions assessed as “black holes” on MRI, reflecting severe neuroaxonal tissue damage [22].

### 2.2. Inflammation

Neuroinflammation is a critical pathological process in MS, both in the acute stages and beyond it. In MS, chronic inflammation is mainly restricted to the CNS, behind the closed blood–brain barrier [37,38]. From its earliest stages, neuroinflammation contributes to neurodegeneration and the failure of remyelination in MS [13,39,40,41]. However, the characterization and monitoring of chronic inflammation in patients is limited, mainly because of the relatively low sensitivity of conventional clinical imaging techniques. Novel PET tracers have provided in vivo validation for the role of microglial activation in chronic inflammation in MS, thus allowing for a sensitive and specific tool for detecting and phenotyping chronic inflammation. 

#### 2.2.1. PET Imaging of Activated Macrophages and Microglia

The 18kDa translocator protein (TSPO) is a transmembrane domain protein located primarily in the outer mitochondrial membrane of cells and is expressed predominantly on glial cells. TSPO was first discovered in 1977 as the binding site of benzodiazepines [42]. TSPO expression is upregulated in several pathophysiological states, including chronic neuroinflammation in MS [43,44,45,46]. As in the case of myelin, several tracers have been used, differing in their kinetics and specificity for TSPO [47,48,49,50,51,52,53,54].

#### 2.2.2. Characterization of Chronic Inflammation Inside and Outside MS Lesions

Studying TSPO PET has confirmed the role that inflammation plays in lesion evolution, revealing that inflammation is present since the early stages of the disease, even before lesions can be identified on MRI [51]; more specifically, activated macrophages and microglia concentrate around MS lesions and also outside lesions in the NAWM and grey matter [50,51,52,53,55]. TSPO PET has revealed heterogeneity between chronic MS lesions in the inflammatory distribution and milieu in lesions that otherwise appear indistinguishable in T2w MRI images [56,57]. This allows for differentiating chronic active lesions with ongoing inflammation from inactive lesions. Furthermore, an increased TSPO uptake was found in MS lesions known as “black holes” in T1w MRI images. These lesions are thought to represent non-inflammatory foci with severe and irreversible neuroaxonal damage. The presence of innate immune cells in a subset of these lesions undercuts the current interpretation of MRI-based lesions classification, revealing that inflammation in and around chronic lesions is much more expansive than previously believed [52,58].

#### 2.2.3. Reinforcing the Association of Chronic Neuroinflammation and Disease Progression

The increased TSPO tracer uptake in NAWM and perilesional WM at the baseline can successfully predict later disability independent of relapse activity [47,59]. Furthermore, an increased TSPO uptake occurs both in NAWM and in WM lesions and correlates with several conventional MRI outcome measures of disease progression, such as an enlarged lesion and brain atrophy [49,50,53,60]. Similar results have been shown in the TSPO uptake in the grey matter [48].

These findings support the role of chronic inflammation as a driver of clinical deterioration, where the activation of innate immunity in the early stages of the disease can affect long-term progression, independent of later relapses. When assessing these results through the lens of this review, this is a convincing demonstration of the potential utility of TSPO PET to predict silent clinical progression in patients with MS.

#### 2.2.4. TSPO PET as a Biomarker for Novel Drug Development

TSPO uptake was used to measure the effect of two immunomodulating multiple sclerosis drugs. TSPO PET was used to assess changes in microglial activation following treatment with natalizumab and fingolimod. A reduced microglial activation was seen in NAWM and at the rim of chronic lesions following one year of natalizumab treatment [54]. Similarly, fingolimod’s effect on microglial activation was also successfully assessed using TSPO PET [61]. TSPO PET is used as a secondary measure of brain activity for an ongoing phase 2 clinical trial evaluating a drug called VX15/2503 for Huntington’s disease (ClinicalTrials.gov ID-NCT02481674).

#### 2.2.5. Promising Novel PET-Tracers of Neuroinflammation in the Development

Additional PET tracers of neuroinflammation in MS exist, though most are still far behind the applicability and real-world potential of TSPO [62]. B cells can be detected in the CNS using rituximab PET tracers [63], allowing for an in vivo investigation of B cell behavior in MS, potentially targeting them as a therapeutic target. The gamma-aminobutyric acid (GABA) receptor density may help to uncover the relationship between the GABAergic system and inflammation in patients with MS. Immune-driven GABAergic activation may be present in MS [64]. Other targets, such as vascular cell adhesion molecule 1 (VCAM-1) expression and P-glycoprotein transporters, exist, but relevant, up-to-date data that outline their potential for MS research has yet to be carried out [65,66].

### 2.3. Neurodegeneration

Assessing neurodegeneration in MS plays a critical part in understanding disease progression, prognosis, and treatment [67]. In MS, neurodegeneration, neuroinflammation, and demyelination are intimately intertwined but highly variable between patients and lesions. Painting these processes as either consequential to one another or independently existing has yet to be resolved. 

Neurodegeneration appears early in the disease and is already detectable in its preclinical stages [68]. Conventional MRI sequences cannot adequately distinguish between these key pathological processes. This is especially true for neuroaxonal damage and demyelination, primarily because of the co-occurrence of these two processes and their overlapping pathology. Axonal pathology primarily represents irreversible damage, preventing successful remyelination if present. Thus, identifying demyelinating lesions with preserved tissue integrity is an essential first step when assessing repair potential. With PET imaging, neurodegeneration is embellished as a reduction in or destruction of a particular aspect of neuronal function. As such, PET imaging is an attractive tool for investigating the molecular mechanism underlying neurodegeneration and its clinical impact on disease progression. 

#### 2.3.1. Fluorodeoxyglucose (FDG)-PET Is a Robust but Nonspecific Marker of Neurodegeneration

Finding suitable tracers to track neurodegeneration in MS has a long track record, starting with the general quantification of F-FDG [69]. This approach provides an indirect measure of neuronal function by quantifying glucose consumption in the CNS, to which, neurons are the main contributors. With neurodegeneration, the FDG uptake declines, serving as a biomarker for neuronal loss. FDG PET has been used to explore therapeutic effects in several diseases, including Alzheimer’s disease, Huntington’s disease, Parkinson’s disease, and schizophrenia [70,71,72,73,74]. In MS, the reduced uptake of FDG in several regions correlated with both the disease duration and severity [75,76]. The interpretation of FDG-PET as a marker of neurodegeneration should be made cautiously because glucose uptake is an indirect and nonspecific marker of axonal integrity, and is influenced by inflammation and impaired metabolism.

#### 2.3.2. Investigating Synapse Dynamics In Vivo

In post-mortem studies of MS brains, a significant synaptic density loss is seen, both in demyelinated lesions and normal-appearing grey matter. In contrast, axonal loss is mainly observed in demyelinated areas. Synaptic vesicle glycoproteins 2A (SV2A) are involved in neurotransmitter transportation in the CNS and have been linked to several neuronal disorders, such as epilepsy, schizophrenia, Alzheimer’s disease, and Parkinson’s disease [77]. Discovered initially as the binding site of the anti-epileptic drug levetiracetam, SV2A is regarded as a biomarker of synaptic density and serves as a surrogate marker of neural integrity. Several SV2A PET tracers differing slightly in their kinetics, imaging properties, and uptake profiles [78] have been used as markers of neurodegeneration in various neurological and psychiatric conditions [79,80,81,82,83]. A clinical trial investigating the possibility of using SV2A-PET as a marker of synaptic density in progressive MS compared to relapsing-remitting MS (RRMS) and healthy controls is underway, and is the first to gauge SV2A’s tie to MS patients (ClinicalTrials.gov ID–NCT04634994).

Flumazenil binds to the benzodiazepine site on GABAa receptors in the CNS. Flumazenil-PET serves as a marker for benzodiazepine receptor density. The tracer was investigated as a marker of neuronal dysfunction in amyotrophic lateral sclerosis (ALS), epilepsy, and essential tremor [84,85,86], showing variable results but overall supporting flumazenil’s utility. In MS, a proof of concept study found a reduced flumazenil uptake in a demyelinated optic tract lesion in a patient with homonymous hemianopia, reflecting a reduced density of GABAa receptors in the affected brain tracts [87]. An in vivo study comparing flumazenil-PET to PK-PET, a TSPO ligand, demonstrated that GABAa receptors are upregulated in MS patients. This upregulation is associated with the innate immune response in the cortex, suggesting that GABAergic abnormalities may be present in MS and that they can serve as a target for MS follow-up [64]. Other studies showed a reduction in the GABAa receptor density in MS patients compared to the healthy control [88]. Taking these findings together suggests that flumazenil’s role in MS clinical trials and practice has yet to be determined and that further studies are needed.

### 2.4. Summary

PET tracers provide an in vivo insight into principal pathophysiological mechanisms associated with silent disease progression. PET-tracers offer better radiological–pathological correlation when compared to traditional MRI sequences. Directly interrogating molecular and cellular alternation with PET is vital when exploring a complex disease such as MS. Originally, one of the main drawbacks of PET was its low spatial resolution compared to MRI. However, combing PET with MRI continues to evolve, allowing for an improved resolution and better representation of the pathophysiology behind MS. New mathematical models are transforming PET acquisition, providing better image quality [89]. Like many other disciplines, the use of generative adversarial networks (GANs) has forced its way into PET-MRI, improving its utility as a predictive biomarker by allowing for extrapolation from one imaging modality to another [90,91].

## 3. Sodium MRI (NaMRI)

Traditional MRI cannot project metabolism and cellular physiology information, making it a weak biomarker of myelin regeneration and neuroaxonal loss. As a result, MRI is a poor barometer of recovery. Conventional MRI relays the abundance of hydrogen nuclei (i.e., proton, H+) in water to generate images. Following hydrogen protons (H+), sodium possesses the most favorable nuclear properties and the largest in vivo concentrations, enabling its use as a signal source for NaMRI. First presented in 1983 as a sensitive tool that shows areas of infarction in a cat stroke model [92], interest in NaMRI has seen many changes. NaMRI is a quantifiable and direct biochemical marker of cell integrity, but sodium’s relatively low tissue concentration has limited its application as a molecular imaging biomarker. Historically, NaMRI images suffered from low signal-to-noise ratios (SNRs), low spatial resolution, and poor tissue contrast. The introduction of high field scanners (≥7 tesla) has dramatically improved SNR, spatial resolution, and reduced scanning time, facilitating the addition of NaMRI to existing “conventional” H-MRI. Indeed, NaMRI is now successfully used to scan different organs, such as the brain, cartilage, heart, kidney, muscle, and breast. These have all reincarnated the image of NaMRI as a modality to invest in [93,94].

Sodium, a principal electrolyte in the body, is essential for maintaining the extracellular volume and transmembrane ion gradient and is particularly important in excitable cells such as neurons [95]. The redistribution of voltage-dependent sodium channels along the demyelinated axons and altered function of Na/K ATPase leads to intracellular sodium accumulation and the breakdown of the transmembrane resting potential [96,97,98]. NaMRI can depict neurodegeneration from its early stages, even before lesions appear on T2w MRI, making it a valuable tool for probing disease pathophysiology. Measuring intracellular and extracellular sodium concentrations with NaMRI allows us to estimate the tissue sodium concentration (TSC), which is the volume fraction weighted mean of the intracellular and the extracellular sodium concentration [99], and reflects sodium homeostasis and cell integrity. As such, it serves as a marker of neuroaxonal tissue integrity and inflammation. 

### 3.1. NaMRI as a Molecular Biomarker of Neuroaxonal Integrity in MS and Other Diseases

The first in vivo example of NaMRI of the human brain was published in 1985 [100], investigating sodium level changes in infarcted and neoplastic tissue. Since then, numerous studies have validated NaMRI’s role in multiple diseases, including stroke, brain tumors, Huntington’s disease, Alzheimer’s disease, migraines, ALS, and epilepsy [101,102,103]. However, the use of NaMRI in MS has a relatively short history. NaMRI studies at 3T and 7T field strengths demonstrated increased TSC in WM MS lesions compared to normal-appearing white and grey matter or the healthy control [104,105]. Further studies confirmed these results, demonstrating increased TSC in MS irrespective of disease subtypes compared with healthy individuals [106,107], validating NaMRI as a reliable tool to decipher MS lesions [108,109,110]. Recent studies shed light on NaMRI’s ability to discriminate between lesions, NAWM, and diffusely abnormal white matter (DAWM). As expected, focal lesions consistently show higher sodium concentrations than NAWM, and NAWM of MS patients show increased sodium values compared to healthy controls. DAWM, depicting the subtle intermediate between NAWM and WMH, showed sodium concentrations resembling NAWM [111]. This means that NaMRI can outline tissue suffering from damage in MS in its earliest stages.

### 3.2. Neuroinflammation and NaMRI

High TSC values were observed in newly formed gadolinium-enhanced lesions [104]. However, tissue integrity is generally preserved in acute MS lesions, suggesting that the increase in TSC values may reflect other pathological changes rather than neurodegeneration. A plausible explanation for this finding is the direct effect of inflammation on cells and the extracellular space (e.g., edema and immune cell infiltrate). 

Eisele et al. further investigated NaMRI’s ability to differentiate between lesions with varying degrees of inflammation [112]. In their study, the sodium concentration was significantly higher in chronic active lesions than in stable or shrinking lesions. The highest concentration was seen in contrast-enhancing lesions, more than in T1 hypointense lesions, and the lowest was observed in T1 isointense lesions. Interestingly, the sodium concentration in hyper scute non-enhancing MS lesions was comparable to the NAWM. This suggests that sodium values can depict active versus chronic inflammation and can identify tissue damage in chronic lesions. In one case study, a centripetal pattern of TSC was observed in an acute enhancing lesion. A similar pattern was described using dynamic contrast-enhanced (DCE) T1w MRI, in which the lesion periphery colocalized with expanding inflammation [113]. Reproducing the results with NaMRI supports its potential as a potential marker of chronic inflammation. This suggests that NaMRI may be sensitive enough to distinguish between lesions according to the extent and pattern of the immune infiltrate, both in the acute and chronic stages. 

A mirrored picture of the destructive lesions with the centripetal TSC and DCE pattern was also published by Eisele et al. The authors described hyperacute lesions with reduced diffusion on conventional H-MRI, but with TSC concentrations comparable to NAWM [114]. Therefore, lesions with similar TSC values to NAWM are lesions with preserved tissue integrity and a possible better outcome. This marks NaMRI as a good candidate for early disease characterization, delineating lesions with potential to gain from early treatment.

### 3.3. Dissecting Effect of Neuroinflammation and Neurodegeneration on NaMRI and Their Association to Meaningful Clinical Outcomes

Two different processes may contribute to the changes outlined above. First, neuroaxonal damage resulting in cell death, edema, and loss of Na/K ATPase action leads to a rise in TSC. On the other hand, a rise in intracellular sodium concentrations (ISC) in the early stages of lesions may reflect metabolic dysfunction of at-risk cells, still evading destruction. NaMRI can discriminate between ISC and extracellular sodium concentrations (ESC), showing areas of increased ISC and decreased ESC in WMH and NAWM of RRMS patients [105]. TSC and ESC were also correlated to lesion volume and EDSS, whereas ISC showed no correlation. ISC was reversely correlated with disease duration, whereas ESC and TSC were correlated directly. This suggests that changes in ISC reflect a compensatory mechanism, more active in the early stages of the disease, becoming less efficient with disease progression. Distinguishing between ISC and ESC clears out certain aspects of the metabolic pathophysiology of MS. A sodium fluid attenuation method similar to the conventional MRI sequence fluid-attenuated inversion recovery (FLAIR) can provide an image weighted towards ISC. This sodium-attenuated form of lesion description might hold potential as a predictor of the future recovery and progression of the disease [115].

### 3.4. Summary

Higher SNR, better resolution, faster acquisition times, and the growing ability to incorporate sodium imaging hardware into existing scanners have prompted the re-evaluation of TSC, ISC, and ESC as viable biomarkers of cellular integrity. Novel studies now look at sodium occupying sites other than the brain as prognostic markers for MS progression [116,117]. Limitations regarding the minimal lesion volume-of-interest still exist [118]. Together with this, NaMRI holds great potential as a marker for the early detection of altered tissue function and a useful prognostic marker for disease progression.

## 4. MRS

Magnetic resonance spectroscopy (MRS) enables an in vivo assessment of biochemical processes, directly measuring various metabolites, neurotransmitters, and the tissue composition of brain matter [119]. In MS, MRS research has examined different metabolites, comparing how they behave in various tissues and patient populations. Starting with a characterization of the metabolic profile of lesions in MS patients [120], research with MRS has explored many metabolites in numerous patient groups and tissues. Some of these metabolites, such as N-acetyl aspartate (NAA), inositols, glutamate, and GABA, present persistent concentration changes in all types of lesions or NAWM compared to healthy controls [121,122,123,124]. Others, such as creatine or choline [125,126], have produced mixed results, not reaching a consensus regarding their biochemical targets and role in MS pathophysiology.

The search for consistency has consequently led to focusing initially on present-day comparisons of metabolite concentrations that can be reliably quantified. Chief among these metabolites is NAA (a marker of neuroaxonal integrity), myo-inositol (a presumed marker of gliosis), and choline (an indicator of an increased membrane turnover and an indirect marker of demyelination).

The most prominent MRS metabolite, NAA, is considered a global marker of neuroaxonal integrity [127]. Decreased NAA is seen in acute and chronic lesions and NAWM of MS patients, and may precede brain atrophy [128]. Thus, incorporating NAA levels as a proxy for lesion detection or disease status theoretically supports the adoption of NAA-MRS as a diagnostic tool. Several other metabolites have served as targets in MRS studies, showing comparable results [129]. Despite these, a low reliability, stemming mainly from methodological challenges, presently holds MRS at bay [130].

Using MRS to predict MS prognosis requires a longitudinal viewpoint. Klauser et al. showed that an increase in some metabolites might precede and predict the appearance of new lesions in MS [131]. Metabolite levels might also help in predicting fatigue levels and disability progression over time [132]. Although with moderate reliability, some metabolites have shown promise in their ability to predict the clinical course of individuals with MS [133]. Ultra-high field scanners (≥7 T) MRS addressed many of the limitations of low-field MRS, resulting in an improved SNR and a better identification and quantification of metabolites [134], enabling the earlier detection of changes in smaller voxels within a shorter timeframe. Thus, it might be attainable to use MRS at the earliest stages of the disease and to utilize it as a sensitive complementary tool for monitoring MS over time [123].

MRS still resides in the realm of biological research, elucidating the biochemical pathophysiology of MS. Contemplating whether a metabolite snapshot can anticipate the disease course in MS is still in debate. MRS acts mainly as a benchmark of “neuroaxonal metabolic health,” which, in a sense, translates to the identification of tissue at risk or targets for remyelination trials. Crossing the threshold into clinical research in the future, MRS will most probably initially serve as an adjunct of lesion detection and indicate NAWM at risk.

## 5. Discussion

Inflammation, neurodegeneration, demyelination, and unsuccessful remyelination are the main pathological drivers of MS progression. All MS therapies successfully mitigate inflammatory relapses, lowering the number of clinical flare-ups and the radiological correlate of active inflammatory disease. Disease-modifying therapies have revolutionized the field of MS, improving patients’ quality of life and lowering clinical disability and disease activity. Unfortunately, controlling acute inflammation only has a modest effect on reducing long-term disability, and chronic clinical disability that characterizes many MS patients can progress parallel and irrespective of the acute inflammation.

Measuring acute inflammation in MS is a staple of disease diagnosis and monitoring. This is performed effectively using standard MRI and molecular adjuncts derived from serum or CSF. Together, these tools enable an accurate acute inflammatory snapshot of patients. Our ability to characterize inflammation is almost taken for granted, engraved in day-to-day clinical practice. Contrary to acute inflammation, silent progression and the chronic disease that accompanies it lacks reliable markers. Standard MRI cannot delve into pathophysiology, and other potential biomarkers have not made the required leap into the robust clinical realm. As of now, we cannot precisely and reliably depict processes on a molecular level, identifying specific cellular culprits that serve as an essential part in disease progression. This lack of biomarkers stalls progress in understanding pathophysiology and the development of novel treatment targets.

In the past, imaging was mainly thought of as an agent of anatomy, and this was also true for MRI of the CNS, traditionally displaying tissue, blood vessels, and the skeleton. However, nuclear magnetic resonance has also enabled the development of several modalities that demonstrate an in vivo layer of information derived from molecular biology. This layer grants us a novel point of view that relies on biology rather than anatomy. In MS, this additional layer is crucial, since collecting specimens from pathology is generally not appropriate. As opposed to fields in medicine, where direct contact with the studied pathology is available, in MS, we are mostly forced to learn from afar, relying on biomarkers derived from various sources.

Recent developments of imaging modalities have covered some of this molecular deficit. Contemporary molecular imaging can display CNS through a molecular prism. This review evaluates modalities whose main advantage lies in their particularity to the pathological substrate, enabling their integration into the clinical trial setup. Tracers used in PET-MRI effectively portray the myelin status and inflammation, and, in the future, might enable a more accurate and specific demonstration of neurodegeneration and recovery. Sodium-based MRI and MRS are more consistent with neuronal damage and are proven tools for assessing tissue health.

Overall, PET is a promising approach for directly assessing myelin and microglia/macrophage dynamics to measure MS lesions repair. This is strongly supported by the fact that PET is already widely used in clinical practice for diagnosing neurodegenerative diseases such as Alzheimer’s disease and Parkinson’s disease. As a result of radiation exposure, PET may not be suited for long-term studies requiring repeated scans. This makes PET a more suitable modality for a comparatively short-term follow-up and scanning, as in clinical trials designed to explore the pro-remyelinating effect of novel therapies. Sodium imaging is an attractive complementary tool for PET, providing an important measure of neuroaxonal health and integrity early in the disease course when the damage may still be reversible. The main limitation of sodium imaging is its relatively low spatial resolution, especially at a low field strength (≤3 T), and the costly specific hardware it requires, thus making it less suited for multicenter trial applications. MRS potentially provides the most direct molecular evaluation from all methods reviewed here, covering all aspects of MS pathophysiology. However, technical challenges, such as a low spatial resolution and inconsistent quantification of metabolites, limit MRS adoption into clinical trials and practice. Ultra-high-field MRI (≥7 T) evades many of these issues by improving the spatial resolution for all modalities and increasing the sensitivity for MRI techniques including MRS [135], as well as reducing the radiation exposure associated with PET-CT.

The incorporation of these tools is pushed onwards by several factors. Sheer technical advances usher in stronger magnets that enable 7T imaging, and the realistic availability of these scanners allows for a higher resolution and finer imaging outputs. The diameter of a lesion that we can portray is not only getting smaller, but its characterization is also shifting. We envision that the progress in molecular imaging modalities we present in this review will enable a more molecular perception of this term in the future, providing a viable complementary tool to standard MRI.
